# Supplementary material for: Cytogenetic signatures favoring metastatic organotropism in colorectal cancer
Source: Nat Commun. 2025 Apr 5;16:3261. doi: 10.1038/s41467-025-58413-1 (PMC11972295; doi:10.1038/s41467-025-58413-1)
Supplement: Supplementary file 1 — Supplementary Information [file 41467_2025_58413_MOESM1_ESM.pdf]

Supplementary Information for

Cytogenetic Signatures Favoring Metastatic Organotropism in Colorectal Cancer

Mariola Monika Golas[1,2,*], Bastian Gunawan[3,9], Angelika Gutenberg[4], Bernhard C. Danner[5], Jan S. Gerdes[3,10], Christine Stadelmann[6], Laszlo Füzesi[3], Torsten Liersch[7], Bjoern Sander[8,*]

[1]Human Genetics, Faculty of Medicine, University of Augsburg, Augsburg, Germany.

[2]Comprehensive Cancer Center Augsburg, University Medical Center Augsburg, Augsburg, Germany.

[3]Institute of Pathology, University Medical Center Göttingen, Göttingen, Germany.

[4]Department of Neurosurgery, Asklepios Hospital Harburg, Hamburg, Germany.

[5]Department of Cardiac, Thoracic and Vascular Surgery, University Medical Center Göttingen, Göttingen, Germany.

[6]Department of Neuropathology, University Medical Center Göttingen, Göttingen, Germany.

[7]Department of General, Visceral and Pediatric Surgery, University Medical Center Göttingen, Göttingen, Germany.

[8]Institute of Pathology, Hannover Medical School, Hannover, Germany.

[9]Present address: Institute of Pathology Northern Hesse, Kassel, Germany.

[10]Present address: Epilepsy Center Hamburg, Evangelical Hospital Alsterdorf, Neurology and Epileptology, Hamburg, Germany.

*Corresponding authors:

M. Monika Golas, monika.golas@med.uni-augsburg.de ORCID 0000-0003-2477-9277

Bjoern Sander, sander.bjoern.pat@mh-hannover.de ORCID 0000-0001-8495-2321

This Supplementary Information contains 4 tables, 7 figures, and 5 references.

Supplementary Table 1: Patient and tumor characteristics of the CRCTropism cohort.

**Patient and tumor characteristics**	
Total number of patients*	191
Sex of CRC patients (females : males)	68 : 123
Mean age at cancer diagnosis (standard deviation)	
Diagnosis of primary CRC	61.4 years (±8.3 years)
Diagnosis of liver metastasis	60.6 years (±10.1 years)
Diagnosis of lung metastasis	61.6 years (±9.4 years)
Diagnosis of brain metastasis	64.1 years (±9.8 years)
Total number of tumors analyzed	314
Primary CRC	80
Liver metastasis	117
Lung metastasis	78
Brain metastasis	39
Primary tumor – metastasis pairs*	97

*, patients may present with multiple tumor manifestations.

Supplementary Table 2: Chromosomal aberration analysis of the CRCTropism cohort. Kruskal-Wallis tests were performed, followed by Dunn post-hoc tests (liver, n = 90; lung, n = 68; brain, n = 33). All p-values are adjusted for multiple testing using the Benjamini-Hochberg method. Significant p-values (p_{adjust} < 0.05) are bolded. CI, chromosomal imbalance.

CI type	p_{adjust}-value (Kruskal-Wallis test)	combination	p_{adjust}-value (Dunn test)
total CIs	**6.10E-23**	liver – lung	**2.46E-03**
		liver – brain	**5.57E-03**
		lung – brain	**1.37E-06**
losses	**5.86E-08**	liver – lung	1.55E-01
		liver – brain	**5.96E-03**
		lung – brain	**3.70E-04**
gains	**1.86E-05**	liver – lung	**1.44E-04**
		liver – brain	**1.75E-02**
		lung – brain	**4.97E-07**
aneuploidies	**1.35E-18**	liver – lung	**2.96E-03**
		liver – brain	**1.31E-02**
		lung – brain	**6.94E-06**

Supplementary Table 3: Patient and tumor characteristics of the CRC patients extracted from the MSK MetTropism cohort[1].

**Patient and tumor characteristics**	
Total number of CRCs	3548
Primary CRC	2401
Liver metastasis	624
Lung metastasis	146
Brain metastasis	22
Other sites or unspecified	355
Primary tumor – metastasis pairs	0
Total number of patients	3548
Sex of CRC patients (females : males)	1618 : 1930
Primary CRC	1069 : 1332
Liver metastasis	289 : 335
Lung metastasis	55 : 91
Brain metastasis	10 : 12
Other sites or unspecified	195 : 160

Supplementary Table 4: Patient characteristics of CRC patients extracted from the TCGA cohort[2,3]. The analysis excluded CRCs belonging to the *POLE* subtype or those with undetermined subtype.

**Patient characteristics**	
Total number of CRCs	449
Primary CRC	449
Liver metastasis	0
Lung metastasis	0
Brain metastasis	0
Primary tumor – metastasis pairs	0
Total number of patients	449
Sex of CRC patients (females : males : NA)	222 : 225 : 2

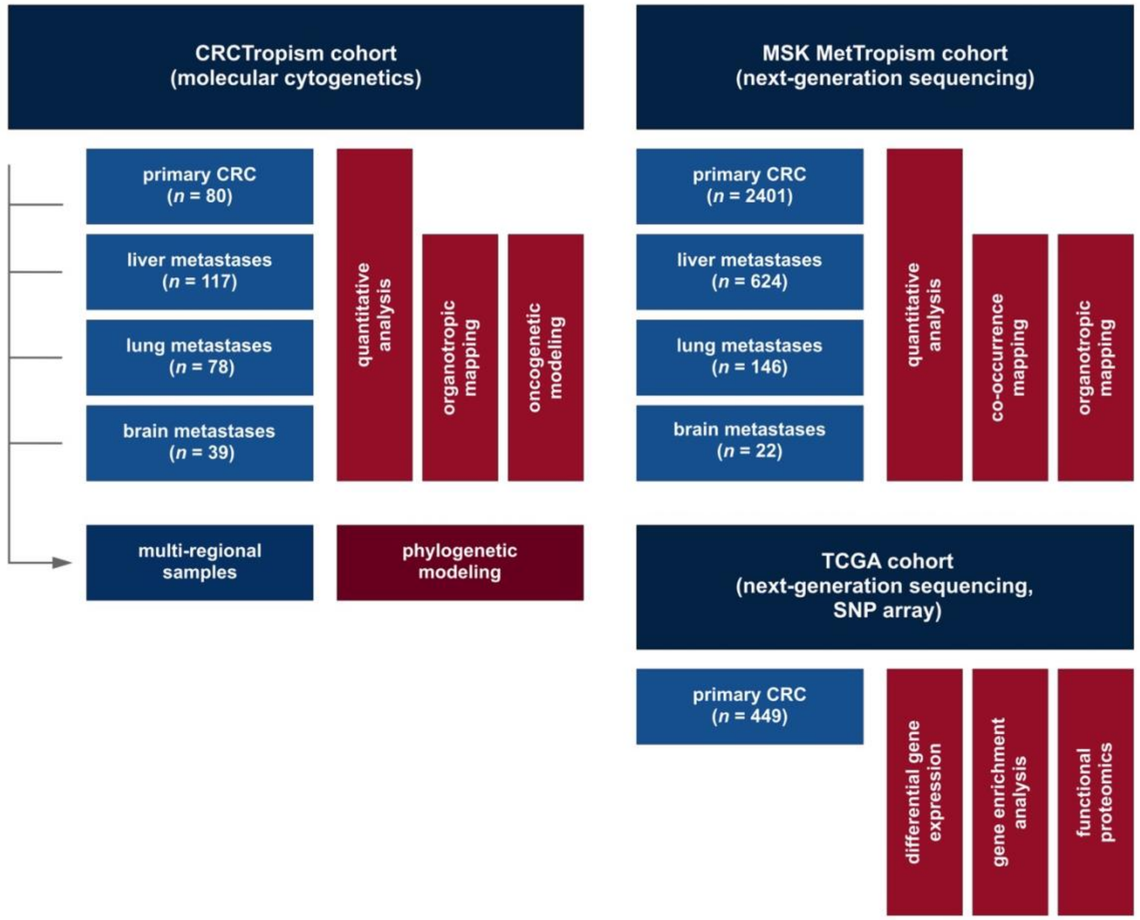

Supplementary Fig. 1: Overview of the analyses performed across three cohorts. Analyzed data include the CRCTropism cohort (our data), the MSK MetTropism cohort[1], and the TCGA cohort[2,3].

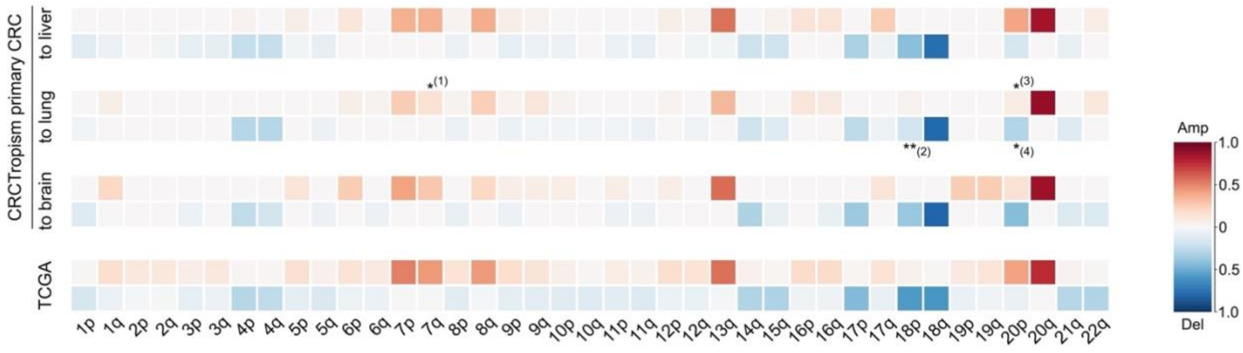

Supplementary Fig. 2: Chromosomal imbalance profile of primary CRC of the CRCTropism and TCGA cohorts. The heatmap shows the frequency of chromosomal arm aneuploidies. The color scale on the right represents the rates. Primary CRC were stratified into subgroups based on reported metastatic dissemination. A given primary CRC may be considered in multiple groups. For comparative purposes, the aneuploidy frequencies derived from the TCGA dataset, as previously reported[4] (n = 585), are also presented. Fisher exact tests with Benjamini-Hochberg correction of multiple testing were used. (1) $p_{adjust} = 0.013$, n = 620; (2) $p_{adjust} = 4.17 \times 10^{-3}$, n = 616; (3) $p_{adjust} = 0.013$, n = 612; (4) $p_{adjust} = 0.013$, n = 612 (*, $p_{adjust} < 0.05$; **; $p_{adjust} < 0.01$). Source data are provided as a Source Data file.

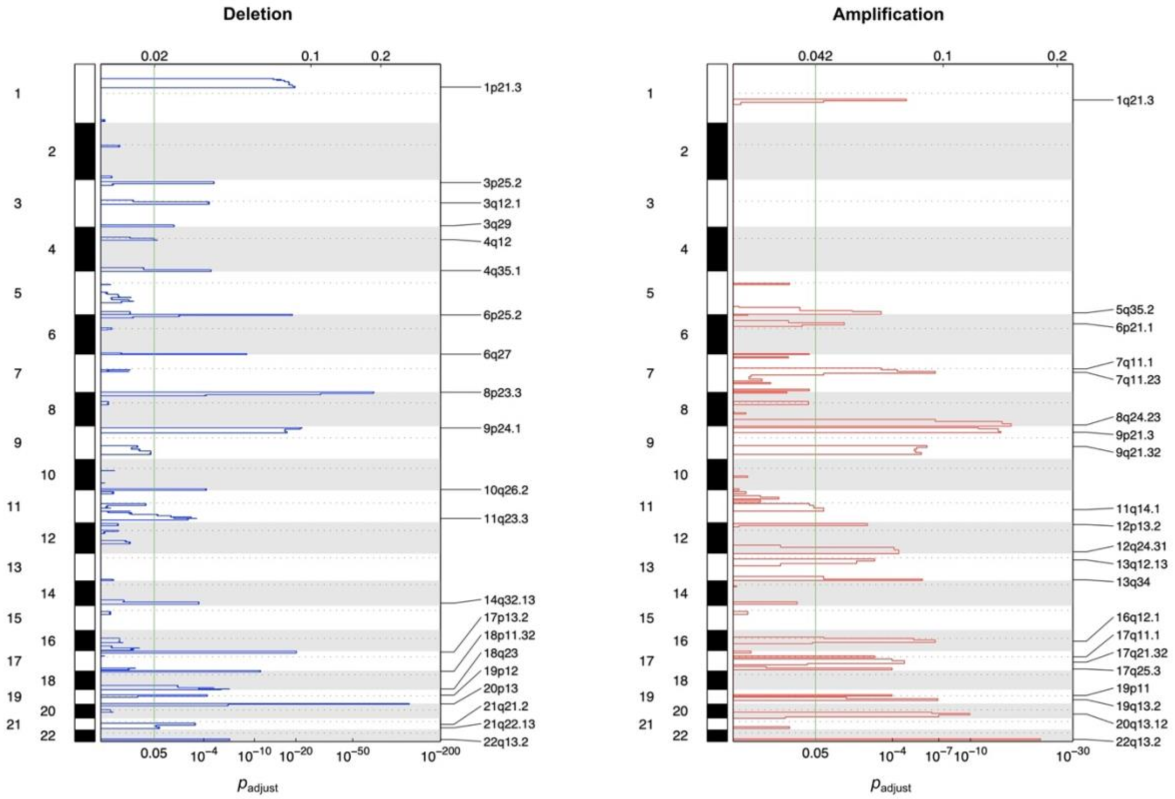

Supplementary Fig. 3: GISTIC-derived deletion and amplifications plots of the CRCTropism cohort.

Autosomal chromosomes are indicated on the left side of the plots.

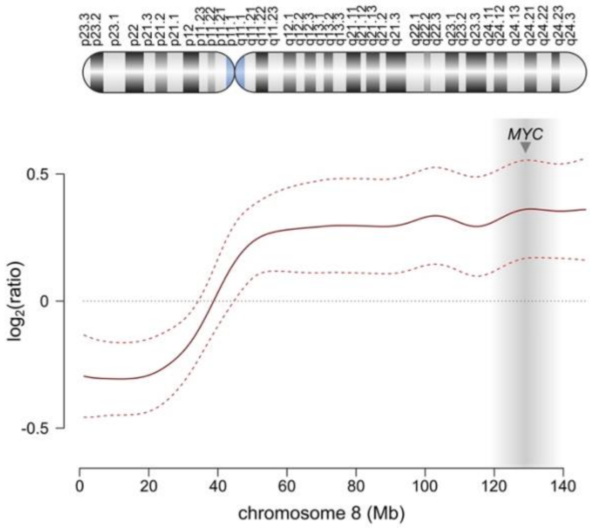

Supplementary Fig. 4: Chromosomal imbalance profile of chromosome 8 in the CRCTropism cohort. The solid line depicts the mean signal ratio ($\log_2$) across the chromosome, with dashed lines representing one standard deviation above and below the mean. The horizontal dotted line indicates the baseline for no imbalance. The breakpoints are predominantly observed in the proximal region of the chromosomal 8p arm, in line with previous data[5]. A notable increase in the signal ratio (indicated by the greyish gradient) is seen at the *MYC* locus (indicated by arrowhead). For orientation, an ideogram of chromosome 8 is provided at the top.

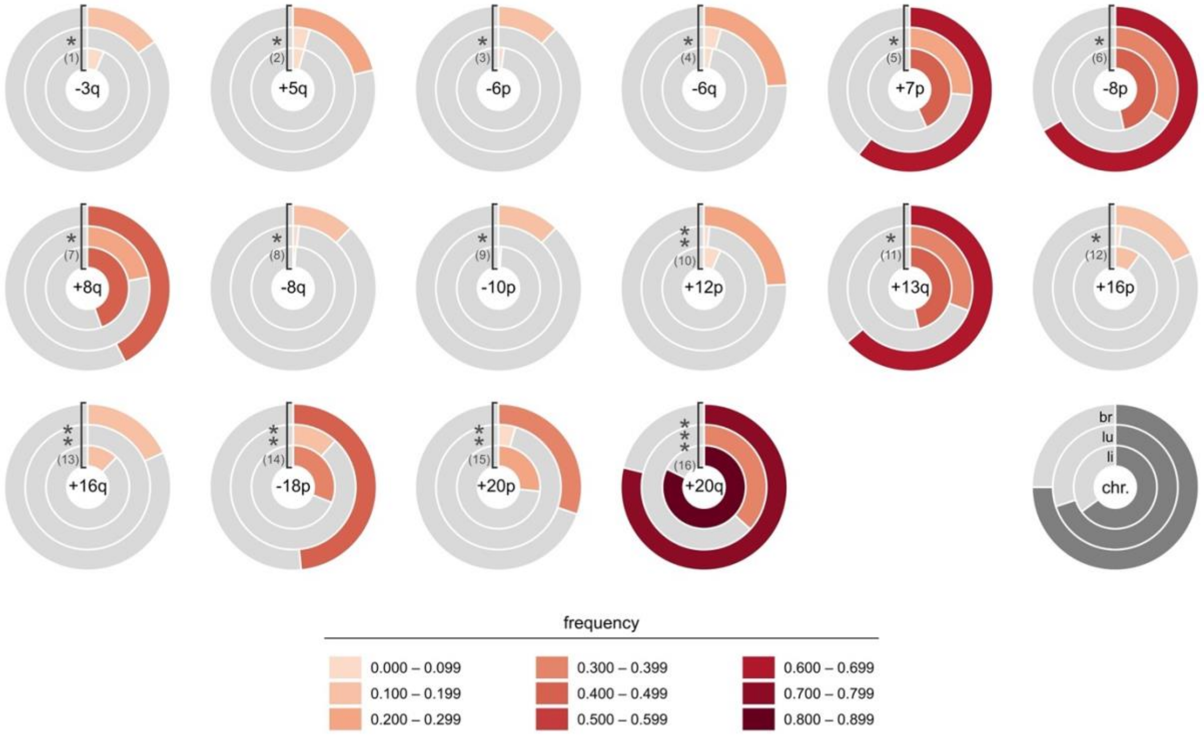

Supplementary Fig. 5: Organotropic chromosomal imbalances (CIs) in CRC metastases from the CRCTropism cohort. The doughnut plots visualize the proportion of metastases (*n* = 90 [liver metastasis], *n* = 68 [lung metastasis], and *n* = 33 [brain metastasis]) harboring specific organotropic CIs across different secondary sites (li - liver, lu - lung, br - brain, chr. - chromosomal arm). Selected plots are shown in Fig. 2 of the main manuscript. The color coding indicates the frequency of each CI observed in the metastasis types. Two-sided Fisher exact tests were performed, with *p*-values adjusted for multiple comparisons using the Benjamini-Hochberg method (*, $p_{adjust} < 0.05$; **, $p_{adjust} < 0.01$; ***, $p_{adjust} < 0.001$; (1) $p_{adjust} = 0.025$; (2) $p_{adjust} = 0.038$; (3) $p_{adjust} = 0.038$; (4) $p_{adjust} = 0.011$; (5) $p_{adjust} = 0.025$; (6) $p_{adjust} = 0.038$; (7) $p_{adjust} = 0.041$; (8) $p_{adjust} = 0.044$; (9) $p_{adjust} = 0.025$; (10) $p_{adjust} = 7.80 \times 10^{-3}$; (11) $p_{adjust} = 0.038$; (12) $p_{adjust} = 0.038$; (13) $p_{adjust} = 7.80 \times 10^{-3}$; (14) $p_{adjust} = 4.85 \times 10^{-3}$; (15) $p_{adjust} = 4.22 \times 10^{-3}$; (16) $p_{adjust} = 3.08 \times 10^{-7}$). Source data are provided as a Source Data file.

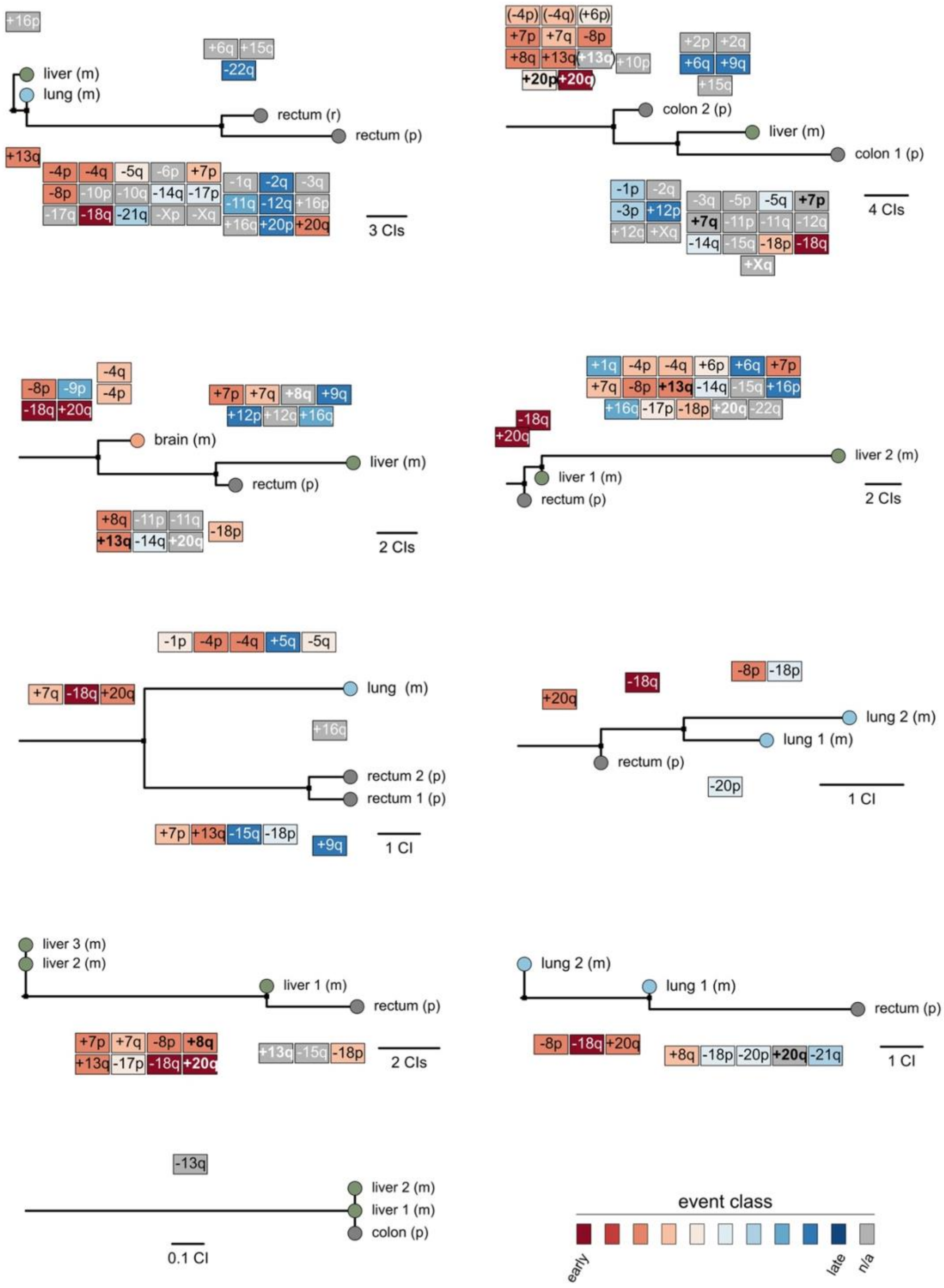
+16p
+6q +15q
-22q
liver (m)
lung (m)
rectum (r)
rectum (p)
+13q
-4p -4q -5q -6p +7p
-8p -10p -10q -14q -17p
-17q -18q -21q -Xp -Xq
-1q -2q -3q
-11q -12q +16p
+16q +20p +20q
3 CIs
(-4p) (-4q) (+6p)
+7p +7q -8p
+8q +13q +13q +10p
+20p +20q
+2p +2q
+6q +9q
+15q
colon 2 (p)
liver (m)
colon 1 (p)
-1p -2q
-3p +12p
+12q +Xq
-3q -5p -5q +7p
+7q -11p -11q -12q
-14q -15q -18p -18q
+Xq
4 CIs
-8p -9p
-18q +20q
-4q
-4p
+7p +7q +8q +9q
+12p +12q +16q
brain (m)
liver (m)
rectum (p)
+8q -11p -11q
+13q -14q +20q
-18p
2 CIs
+1q -4p -4q +6p +6q +7p
+7q -8p +13q -14q -15q +16p
+16q -17p -18p +20q -22q
-18q
+20q
liver 2 (m)
liver 1 (m)
rectum (p)
2 CIs
-1p -4p -4q +5q -5q
+7q -18q +20q
lung (m)
+16q
rectum 2 (p)
rectum 1 (p)
+7p +13q -15q -18p
+9q
1 CI
-8p -18p
-18q
+20q
lung 2 (m)
lung 1 (m)
rectum (p)
-20p
1 CI
liver 3 (m)
liver 2 (m)
liver 1 (m)
rectum (p)
+7p +7q -8p +8q
+13q -17p -18q +20q
+13q -15q -18p
2 CIs
lung 2 (m)
lung 1 (m)
rectum (p)
-8p -18q +20q
+8q -18p -20p +20q -21q
1 CI
-13q
liver 2 (m)
liver 1 (m)
colon (p)
0.1 CI
event class
early
late
n/a

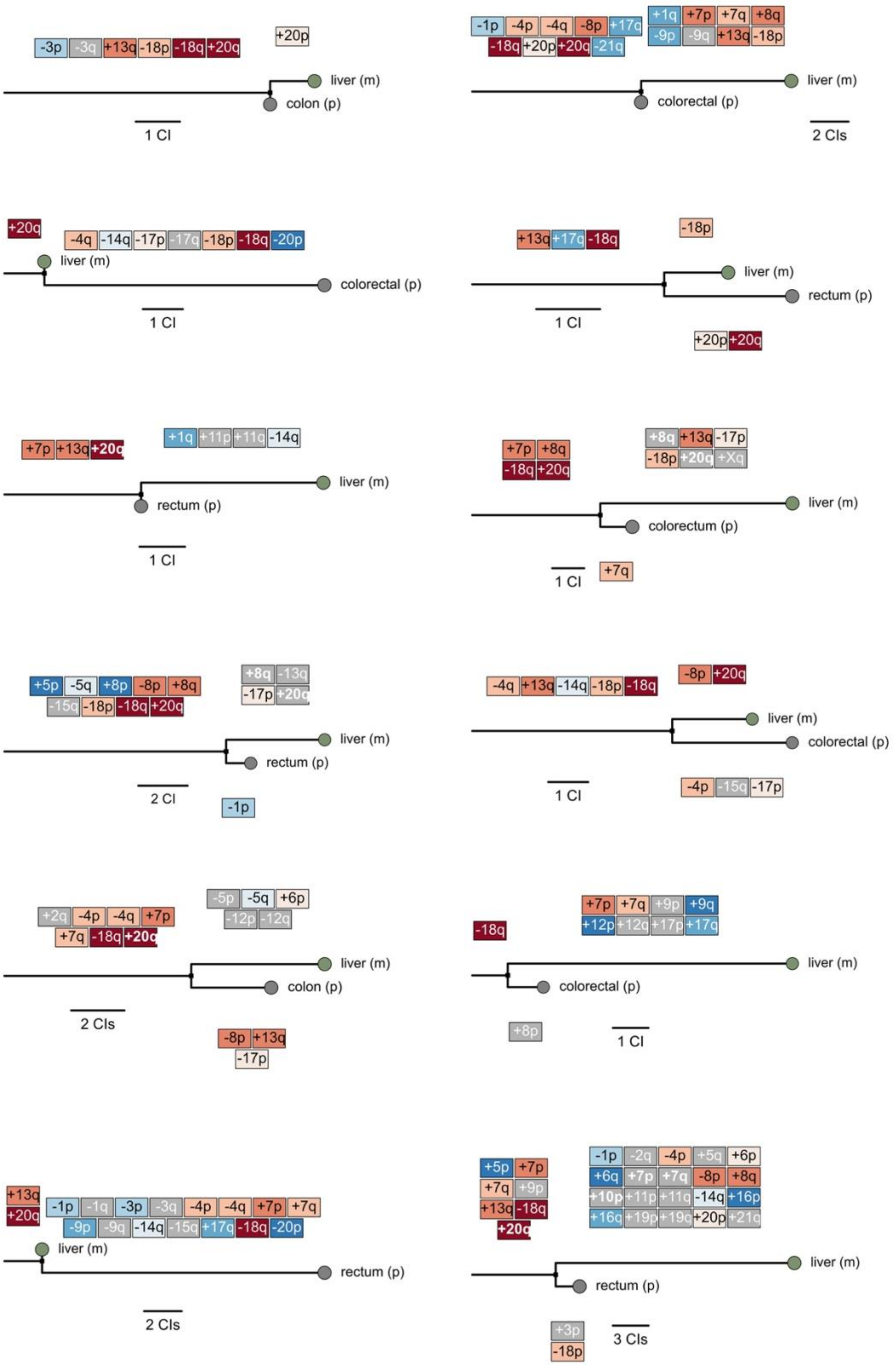
-3p -3q +13q -18p -18q +20q
+20p
liver (m)
colon (p)
1 CI
-1p -4p -4q -8p +17q
-18q +20p +20q -21q
+1q +7p +7q +8q
-9p -9q +13q -18p
liver (m)
colorectal (p)
2 CIs
+20q
-4q -14q -17p -17q -18p -18q -20p
liver (m)
colorectal (p)
1 CI
+13q +17q -18q
-18p
liver (m)
rectum (p)
+20p +20q
1 CI
+7p +13q +20q
+1q +11p +11q -14q
liver (m)
rectum (p)
1 CI
+7p +8q
-18q +20q
+8q +13q -17p
-18p +20q +Xq
liver (m)
colorectum (p)
1 CI
+7q
+5p -5q +8p -8p +8q
-15q -18p -18q +20q
+8q -13q
-17p +20q
liver (m)
rectum (p)
2 CI
-1p
-4q +13q -14q -18p -18q
-8p +20q
liver (m)
colorectal (p)
1 CI
-4p -15q -17p
+2q -4p -4q +7p
+7q -18q +20q
-5p -5q +6p
-12p -12q
liver (m)
colon (p)
2 CIs
-8p +13q
-17p
-18q
+7p +7q +9p +9q
+12p +12q +17p +17q
liver (m)
colorectal (p)
+8p
1 CI
+13q
+20q
-1p -1q -3p -3q -4p -4q +7p +7q
-9p -9q -14q -15q +17q -18q -20p
liver (m)
rectum (p)
2 CIs
+5p +7p
+7q +9p
+13q -18q
+20q
-1p -2q -4p +5q +6p
+6q +7p +7q -8p +8q
+10p +11p +11q -14q +16p
+16q +19p +19q +20p +21q
liver (m)
rectum (p)
+3p
-18p
3 CIs

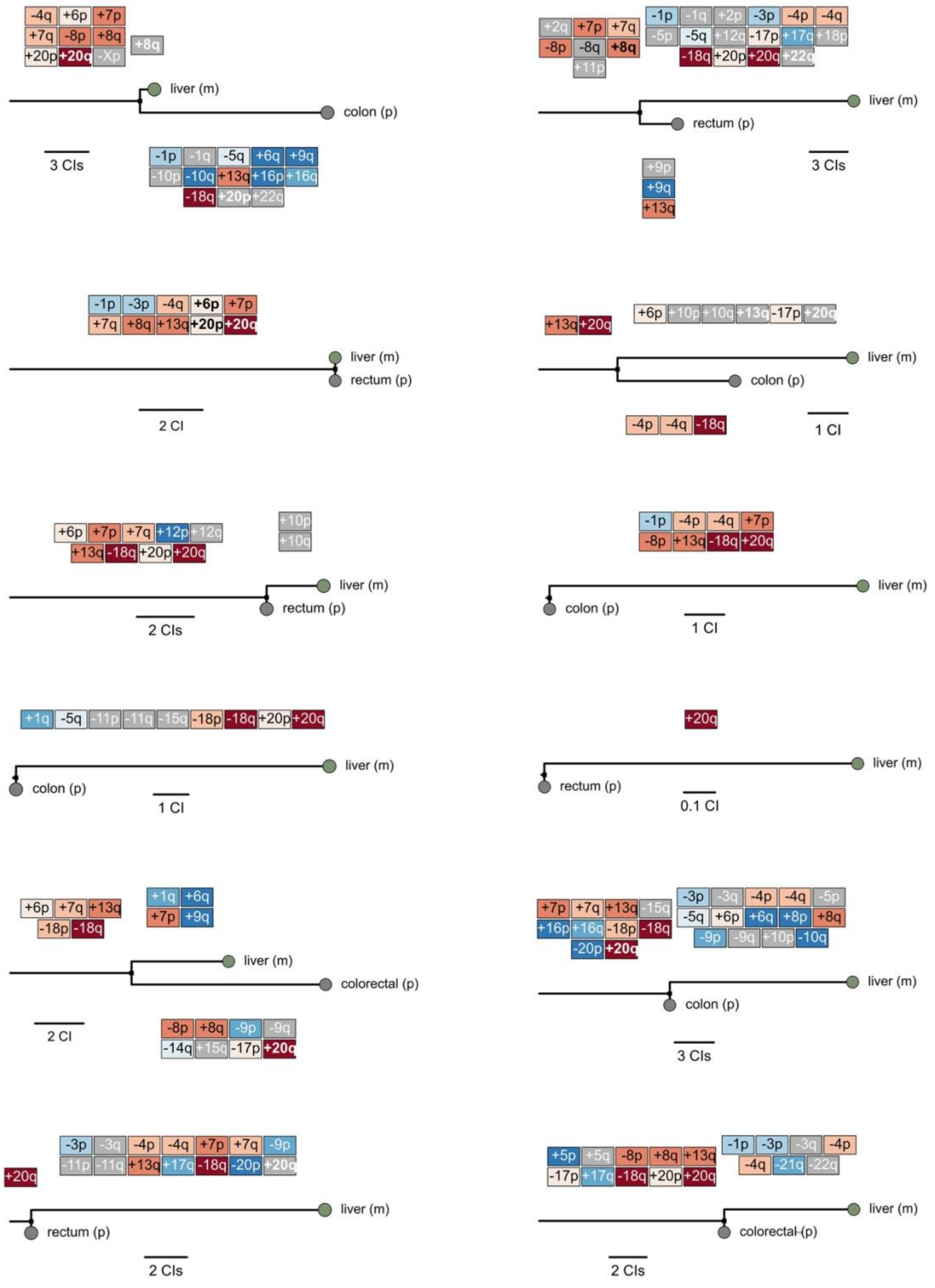
-4q +6p +7p
+7q -8p +8q +8q
+20p +20q -Xp
liver (m)
colon (p)
3 CIs
-1p -1q -5q +6q +9q
-10p -10q +13q +16p +16q
-18q +20p +22q
+2q +7p +7q
-8p -8q +8q
+11p
-1p -1q +2p -3p -4p -4q
-5p -5q +12q -17p +17q +18p
-18q +20p +20q +22q
liver (m)
rectum (p)
3 CIs
+9p
+9q
+13q
-1p -3p -4q +6p +7p
+7q +8q +13q +20p +20q
liver (m)
rectum (p)
2 CI
+13q +20q
+6p +10p +10q +13q -17p +20q
liver (m)
colon (p)
-4p -4q -18q
1 CI
+6p +7p +7q +12p +12q
+13q -18q +20p +20q
+10p
+10q
liver (m)
rectum (p)
2 CIs
-1p -4p -4q +7p
-8p +13q -18q +20q
liver (m)
colon (p)
1 CI
+1q -5q -11p -11q -15q -18p -18q +20p +20q
liver (m)
colon (p)
1 CI
+20q
liver (m)
rectum (p)
0.1 CI
+6p +7q +13q
-18p -18q
+1q +6q
+7p +9q
liver (m)
colorectal (p)
2 CI
-8p +8q -9p -9q
-14q +15q -17p +20q
+7p +7q +13q -15q
+16p +16q -18p -18q
-20p +20q
-3p -3q -4p -4q -5p
-5q +6p +6q +8p +8q
-9p -9q +10p -10q
liver (m)
colon (p)
3 CIs
-3p -3q -4p -4q +7p +7q -9p
-11p -11q +13q +17q -18q -20p +20q
+20q
liver (m)
rectum (p)
2 CIs
+5p +5q -8p +8q +13q
-17p +17q -18q +20p +20q
-1p -3p -3q -4p
-4q -21q -22q
liver (m)
colorectal (p)
2 CIs

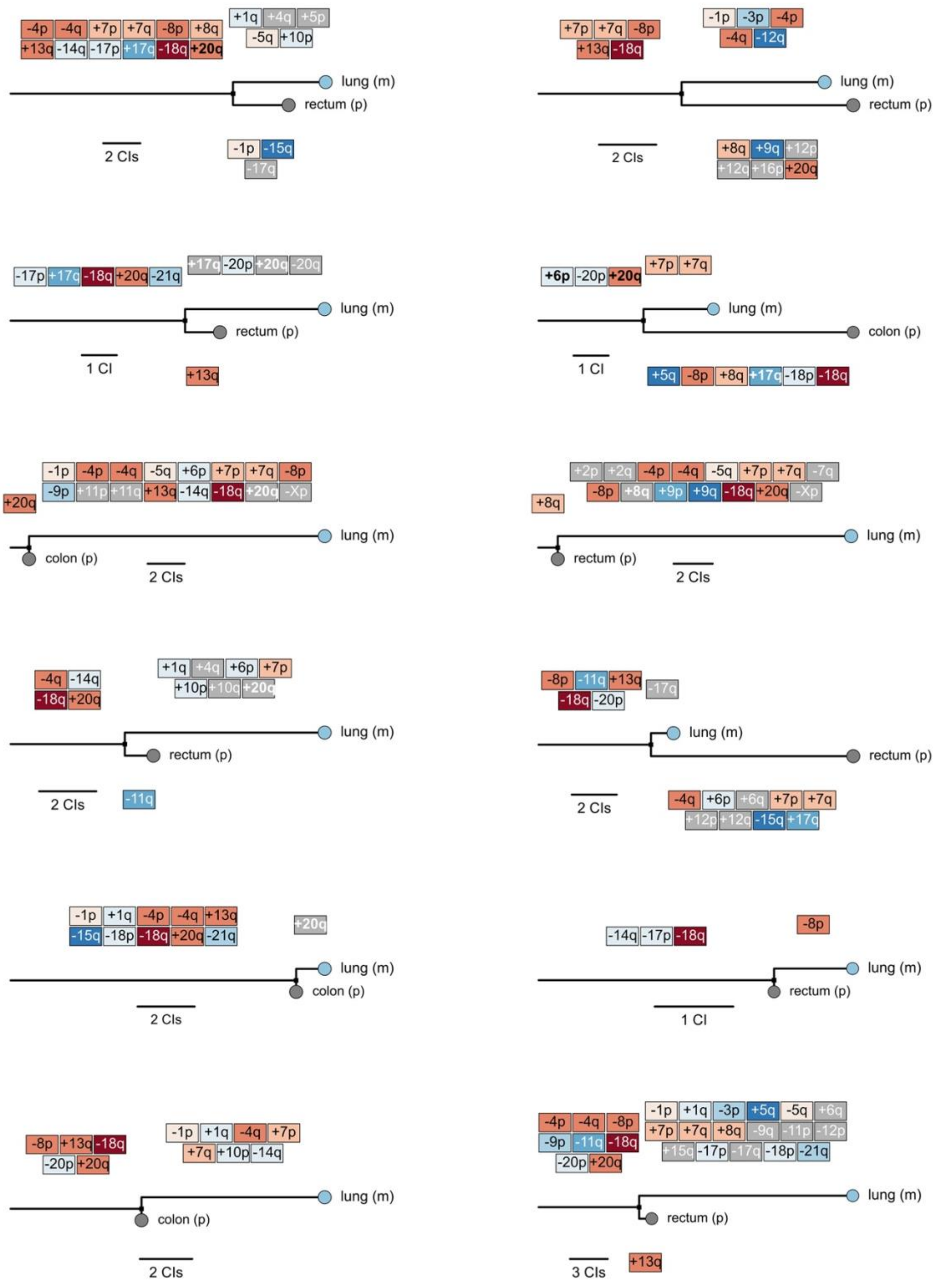

-4p -4q +7p +7q -8p +8q
+13q -14q -17p +17q -18q +20q
+1q +4q +5p
-5q +10p
lung (m)
rectum (p)
2 CIs
-1p -15q
-17q
+7p +7q -8p
+13q -18q
-1p -3p -4p
-4q -12q
lung (m)
rectum (p)
2 CIs
+8q +9q +12p
+12q +16p +20q
-17p +17q -18q +20q -21q
+17q -20p +20q -20q
lung (m)
rectum (p)
1 CI
+13q
+6p -20p +20q
+7p +7q
lung (m)
colon (p)
1 CI
+5q -8p +8q +17q -18p -18q
-1p -4p -4q -5q +6p +7p +7q -8p
-9p +11p +11q +13q -14q -18q +20q -Xp
+20q
lung (m)
colon (p)
2 CIs
+2p +2q -4p -4q -5q +7p +7q -7q
-8p +8q +9p +9q -18q +20q -Xp
+8q
lung (m)
rectum (p)
2 CIs
-4q -14q
-18q +20q
+1q +4q +6p +7p
+10p +10q +20q
lung (m)
rectum (p)
2 CIs
-11q
-8p -11q +13q
-18q -20p
-17q
lung (m)
rectum (p)
2 CIs
-4q +6p +6q +7p +7q
+12p +12q -15q +17q
-1p +1q -4p -4q +13q
-15q -18p -18q +20q -21q
+20q
lung (m)
colon (p)
2 CIs
-14q -17p -18q
-8p
lung (m)
rectum (p)
1 CI
-8p +13q -18q
-20p +20q
-1p +1q -4q +7p
+7q +10p -14q
lung (m)
colon (p)
2 CIs
-4p -4q -8p
-9p -11q -18q
-20p +20q
-1p +1q -3p +5q -5q +6q
+7p +7q +8q -9q -11p -12p
+15q -17p -17q -18p -21q
lung (m)
rectum (p)
3 CIs
+13q

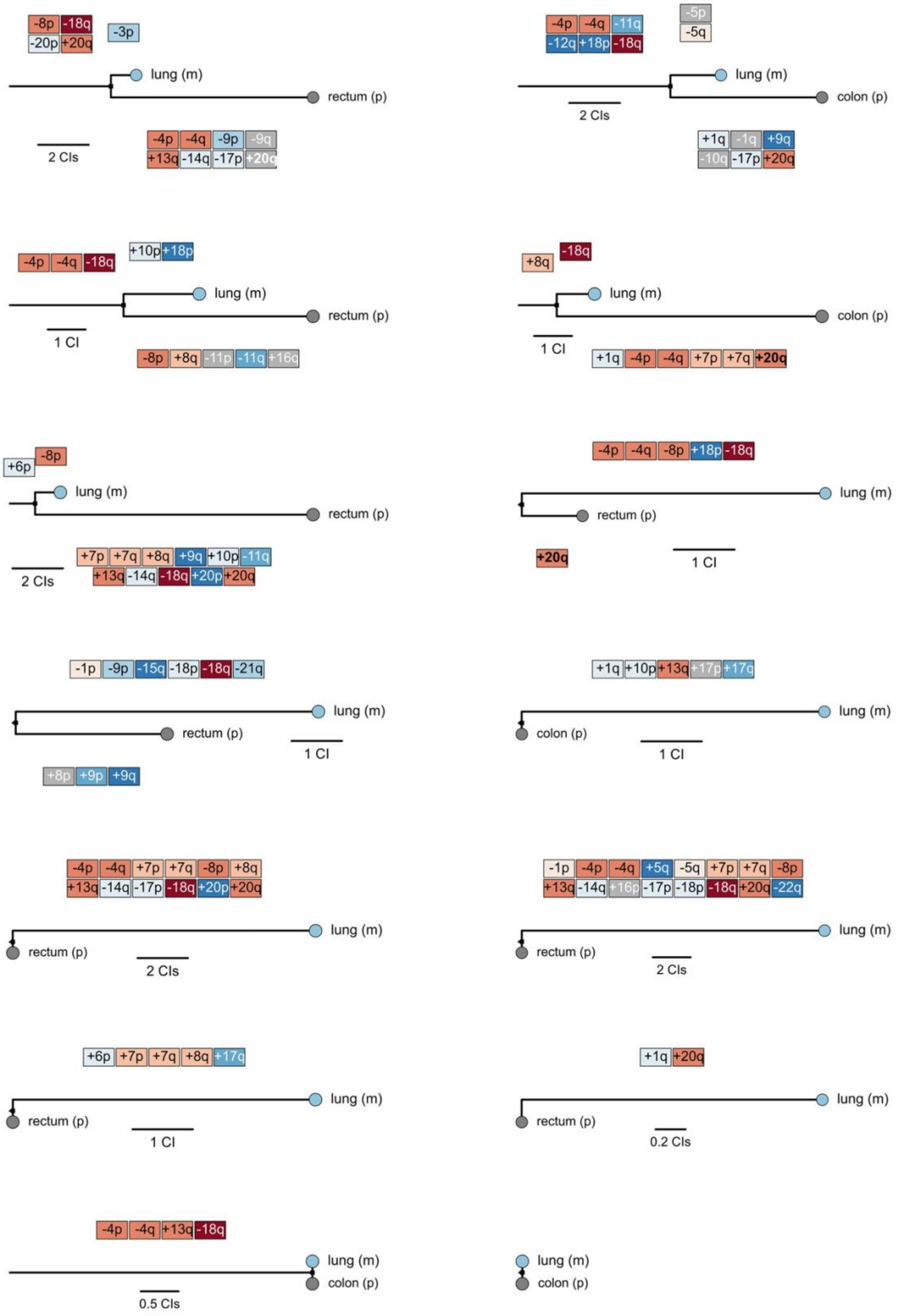

-8p -18q -20p +20q
-3p
lung (m)
rectum (p)
2 CIs
-4p -4q -9p -9q +13q -14q -17p +20q
-4p -4q -11q -12q +18p -18q
-5p -5q
lung (m)
colon (p)
2 CIs
+1q -1q +9q -10q -17p +20q
-4p -4q -18q
+10p +18p
lung (m)
rectum (p)
1 CI
-8p +8q -11p -11q +16q
+8q -18q
lung (m)
colon (p)
1 CI
+1q -4p -4q +7p +7q +20q
+6p -8p
lung (m)
rectum (p)
2 CIs
+7p +7q +8q +9q +10p -11q +13q -14q -18q +20p +20q
-4p -4q -8p +18p -18q
lung (m)
rectum (p)
+20q
1 CI
-1p -9p -15q -18p -18q -21q
lung (m)
rectum (p)
1 CI
+8p +9p +9q
+1q +10p +13q +17p +17q
lung (m)
colon (p)
1 CI
-4p -4q +7p +7q -8p +8q +13q -14q -17p -18q +20p +20q
lung (m)
rectum (p)
2 CIs
-1p -4p -4q +5q -5q +7p +7q -8p +13q -14q +16p -17p -18p -18q +20q -22q
lung (m)
rectum (p)
2 CIs
+6p +7p +7q +8q +17q
lung (m)
rectum (p)
1 CI
+1q +20q
lung (m)
rectum (p)
0.2 CIs
-4p -4q +13q -18q
lung (m)
colon (p)
0.5 CIs
lung (m)
colon (p)

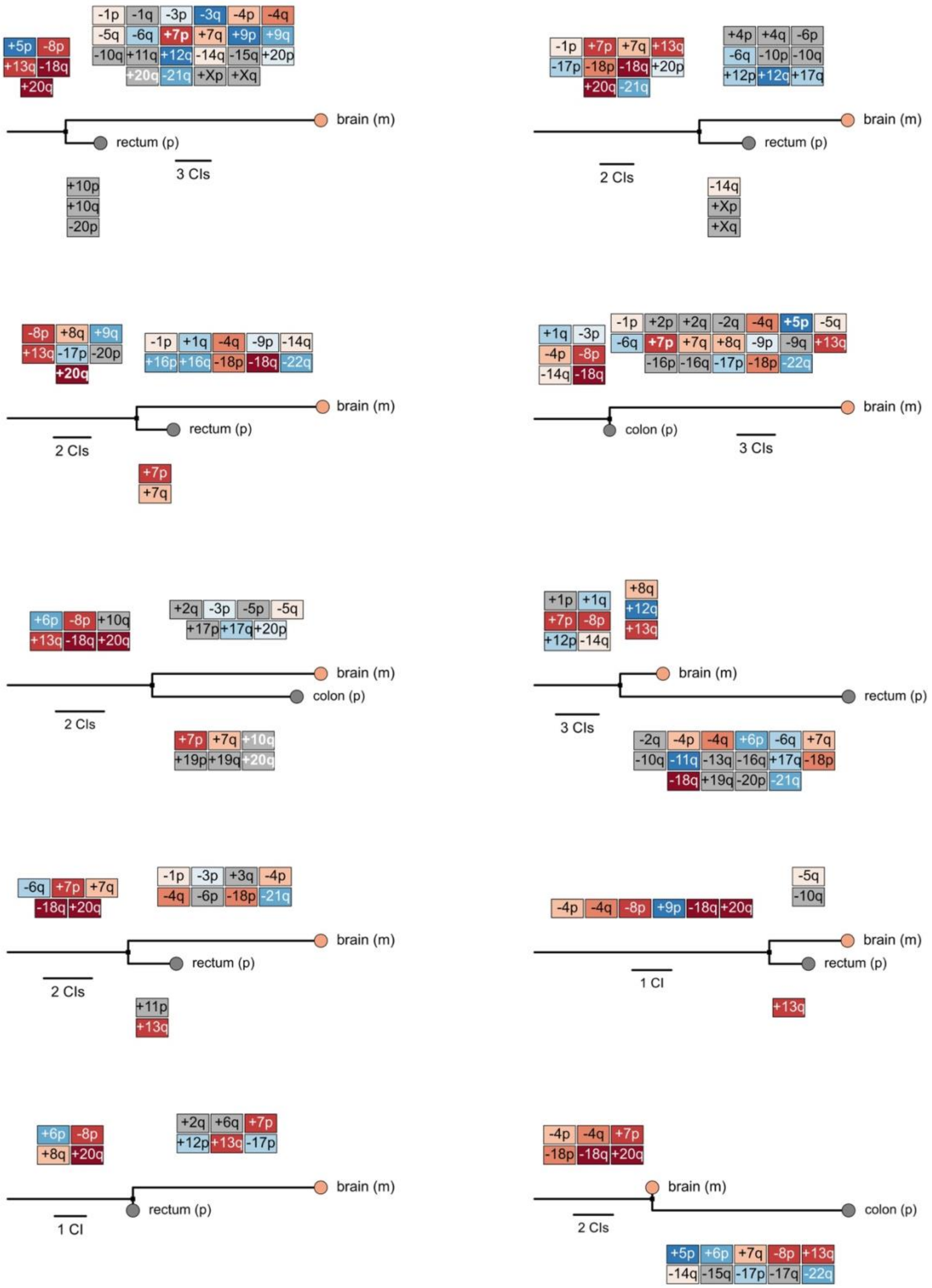
+5p -8p
+13q -18q
+20q
-1p -1q -3p -3q -4p -4q
-5q -6q +7p +7q +9p +9q
-10q +11q +12q -14q -15q +20p
+20q -21q +Xp +Xq
brain (m)
rectum (p)
3 CIs
+10p
+10q
-20p
-1p +7p +7q +13q
-17p -18p -18q +20p
+20q -21q
+4p +4q -6p
-6q -10p -10q
+12p +12q +17q
brain (m)
rectum (p)
2 CIs
-14q
+Xp
+Xq
-8p +8q +9q
+13q -17p -20p
+20q
-1p +1q -4q -9p -14q
+16p +16q -18p -18q -22q
brain (m)
rectum (p)
2 CIs
+7p
+7q
+1q -3p
-4p -8p
-14q -18q
-1p +2p +2q -2q -4q +5p -5q
-6q +7p +7q +8q -9p -9q +13q
-16p -16q -17p -18p -22q
brain (m)
colon (p)
3 CIs
+6p -8p +10q
+13q -18q +20q
+2q -3p -5p -5q
+17p +17q +20p
brain (m)
colon (p)
2 CIs
+7p +7q +10q
+19p +19q +20q
+1p +1q
+7p -8p
+12p -14q
+8q
+12q
+13q
brain (m)
rectum (p)
3 CIs
-2q -4p -4q +6p -6q +7q
-10q -11q -13q -16q +17q -18p
-18q +19q -20p -21q
-6q +7p +7q
-18q +20q
-1p -3p +3q -4p
-4q -6p -18p -21q
brain (m)
rectum (p)
2 CIs
+11p
+13q
-5q
-10q
-4p -4q -8p +9p -18q +20q
brain (m)
rectum (p)
1 CI
+13q
+6p -8p
+8q +20q
+2q +6q +7p
+12p +13q -17p
brain (m)
rectum (p)
1 CI
-4p -4q +7p
-18p -18q +20q
brain (m)
colon (p)
2 CIs
+5p +6p +7q -8p +13q
-14q -15q -17p -17q -22q

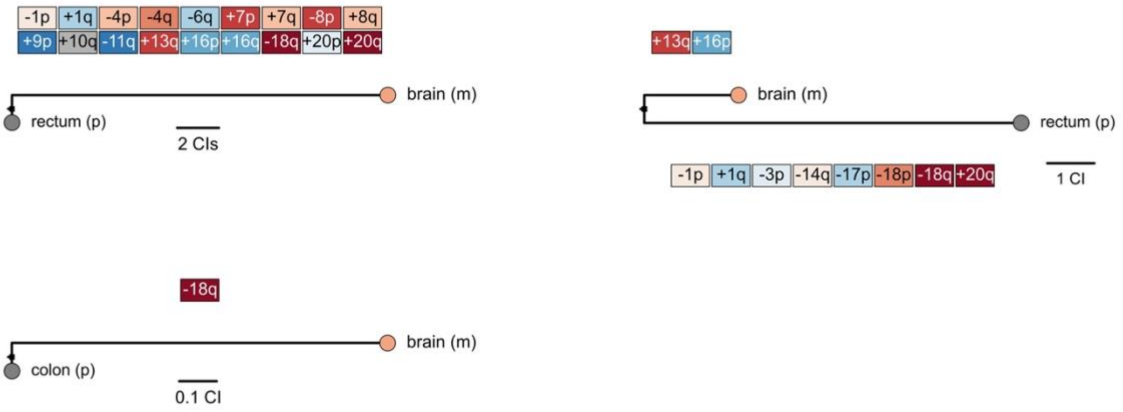

Supplementary Fig. 6: Patient-specific phylogenetic trees derived from multi-sample cytogenetic data in the CRCTropism cohort. Phylogenetic trees were reconstructed for individual CRC patients using multi-sample cytogenetic data from the CRCTropism cohort. CI events are color-coded according to their temporal classification (early to late) as determined by oncogenetic tree analysis (see Fig. 6 for corresponding oncogenetic trees). For additional reconstructions, refer to Fig. 7 in the main manuscript. Source data are provided as a Source Data file.

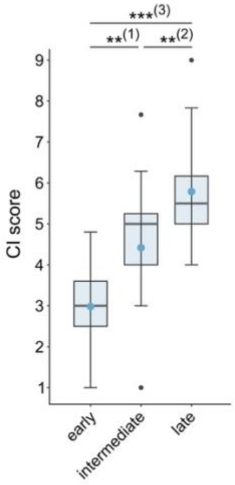

Supplementary Fig. 7: Classification of CI events based on patient-specific phylogenetic trees, as observed in the CRCTropism cohort. The plot illustrates the distribution of CI scores across early, intermediate, and late temporal categories, as derived from the phylogenetic trees of individual patients (n = 13). The CI event class is derived from the oncogenetic trees (see Fig. 6 in the main manuscript). Data are visualized as box-and-whisker-plots (thick line, median; box edges, Q1/Q3; whiskers, 1.5× IQR, black points, individual points outside this range). Each plot includes a blue dot indicating the mean score for that category. Linear mixed-effect modeling was employed to assess statistical significance between temporal categories, followed by post-hoc testing with Benjamini-Hochberg correction ((1) $p_{adjust} = 9.27 \times 10^{-3}$; (2) $p_{adjust} = 9.27 \times 10^{-3}$; (3) $p_{adjust} = 2.86 \times 10^{-7}$). Significant differences are indicated by asterisks (**, $p_{adjust} < 0.01$; ***, $p_{adjust} < 0.001$). Source data are provided as a Source Data file.

Supplementary References

1. Nguyen, B. *et al.* Genomic characterization of metastatic patterns from prospective clinical sequencing of 25,000 patients. *Cell* **185**, 563-575 e511 (2022).

2. Liu, Y. *et al.* Comparative Molecular Analysis of Gastrointestinal Adenocarcinomas. *Cancer Cell* **33**, 721-735 e728 (2018).

3. Cancer Genome Atlas Research Network *et al.* The Cancer Genome Atlas Pan-Cancer analysis project. *Nat Genet* **45**, 1113-1120 (2013).

4. Shih, J. *et al.* Cancer aneuploidies are shaped primarily by effects on tumour fitness. *Nature* **619**, 793-800 (2023).

5. Palin, K. *et al.* Contribution of allelic imbalance to colorectal cancer. *Nat Commun* **9**, 3664 (2018).
