# Supplementary material for: Cytogenetic signatures favoring metastatic organotropism in colorectal cancer
Source: Nat Commun. 2025 Apr 5;16:3261. doi: 10.1038/s41467-025-58413-1 (PMC11972295; doi:10.1038/s41467-025-58413-1)
Supplement: Supplementary file 2 — Reporting Summary [file 41467_2025_58413_MOESM2_ESM.pdf]

nature portfolio

Corresponding author(s): Mariola Monika Golas
Bjoern Sander

Last updated by author(s): 20/04/2025

Reporting Summary

Nature Portfolio wishes to improve the reproducibility of the work that we publish. This form provides structure for consistency and transparency in reporting. For further information on Nature Portfolio policies, see our Editorial Policies and the Editorial Policy Checklist.

Statistics

For all statistical analyses, confirm that the following items are present in the figure legend, table legend, main text, or Methods section.

n/a	Confirmed	
☐	☒	The exact sample size (*n*) for each experimental group/condition, given as a discrete number and unit of measurement
☐	☒	A statement on whether measurements were taken from distinct samples or whether the same sample was measured repeatedly
☐	☒	The statistical test(s) used AND whether they are one- or two-sided<br>*Only common tests should be described solely by name; describe more complex techniques in the Methods section.*
☐	☒	A description of all covariates tested
☐	☒	A description of any assumptions or corrections, such as tests of normality and adjustment for multiple comparisons
☐	☒	A full description of the statistical parameters including central tendency (e.g. means) or other basic estimates (e.g. regression coefficient) AND variation (e.g. standard deviation) or associated estimates of uncertainty (e.g. confidence intervals)
☐	☒	For null hypothesis testing, the test statistic (e.g. *F*, *t*, *r*) with confidence intervals, effect sizes, degrees of freedom and *P* value noted<br>*Give P values as exact values whenever suitable.*
☒	☐	For Bayesian analysis, information on the choice of priors and Markov chain Monte Carlo settings
☐	☒	For hierarchical and complex designs, identification of the appropriate level for tests and full reporting of outcomes
☒	☐	Estimates of effect sizes (e.g. Cohen's *d*, Pearson's *r*), indicating how they were calculated

Our web collection on statistics for biologists contains articles on many of the points above.

Software and code

Policy information about availability of computer code

Data collection	Molecular cytogenetics data were collected and processed using Quips software (Vysis,. Downers Grove, IL, U.S.A.). The MSK MetTropism data (Nguyen et al., 2022) were obtained from cbioportal (https://www.cbioportal.org). TCGA data (TCGA, 2013; Liu et al., 2018; Akbani et al., 2014) were downloaded from GDC Portal (https://portal.gdc.cancer.gov) and cBioPortal. For assessment of variant pathogenicity, the resource OncoKB (https://www.oncokb.org) was used.
Data analysis	The following R packages were used in the software suite R (version 4.3.3) for analysis and visualization: stats version 4.3.1, car version 3.1.2, vip version 0.4.1, lmtest version 0.9.40, lme4 version 1.1.35.5, cooccur version 1.3, ggtern version 3.5.0, oncomodel version 1.0, edgeR version 4.0.3, Organism.dplyr version 1.30.1, ggplot2 version 3.5.1, and tidyverse version 2.0.0.<br><br>The following tools were used for analysis and visualization: ASCETS version 1.0, GISTIC version 2.0, GSEA version 20.4.0, ssGSEA version 10.1.0, g:profiler version e111_eg58_p18_f463989d, enrichr (https://maayanlab.cloud/Enrichr/), PHYLIP version 3.697, and FigTree version 1.4.4.

For manuscripts utilizing custom algorithms or software that are central to the research but not yet described in published literature, software must be made available to editors and reviewers. We strongly encourage code deposition in a community repository (e.g. GitHub). See the Nature Portfolio guidelines for submitting code & software for further information.

Data

Policy information about availability of data

All manuscripts must include a data availability statement. This statement should provide the following information, where applicable:

- Accession codes, unique identifiers, or web links for publicly available datasets
- A description of any restrictions on data availability
- For clinical datasets or third party data, please ensure that the statement adheres to our policy

Source data are provided with this paper. The previously published CGH data (1,2) comprise chromosomal imbalance profiles indicating net clonal changes, characterized by regions of chromosomal losses, gains, and amplifications at the chromosomal band level, accompanied by basic demographic information including sex and age. The MSK MetTropism publicly available data (3) used in this study are available in the cBioPortal database (https://www.cbioportal.org) under accession code MSK MetTropsim. The TCGA publicly available data (4-6) used in this study are available at the GDC Portal (https://portal.gdc.cancer.gov) under accession codes TCGA-COAD and TCGA-READ and cBioPortal under accession code Colorectal Adenocarcinoma (TCGA, PanCancer Atlas). The remaining data are available within the Article, Supplementary Information or Source Data file.

References:

1. Danner, B. C. et al. Comparison of chromosomal aberrations in primary colorectal carcinomas to their pulmonary metastases. Cancer Genet 204, 122-128 (2011).
2. Gutenberg, A. et al. High chromosomal instability in brain metastases of colorectal carcinoma. Cancer Genet Cytogenet 198, 47-51 (2010).
3. Nguyen, B. et al. Genomic characterization of metastatic patterns from prospective clinical sequencing of 25,000 patients. Cell 185, 563-575 e511 (2022).
4. Liu, Y. et al. Comparative Molecular Analysis of Gastrointestinal Adenocarcinomas. Cancer Cell 33, 721-735 e728 (2018).
5. Cancer Genome Atlas Research Network et al. The Cancer Genome Atlas Pan-Cancer analysis project. Nat Genet 45, 1113-1120 (2013).
6. Akbani, R. et al. A pan-cancer proteomic perspective on The Cancer Genome Atlas. Nat Commun 5, 3887 (2014).

Research involving human participants, their data, or biological material

Policy information about studies with human participants or human data. See also policy information about sex, gender (identity/presentation), and sexual orientation and race, ethnicity and racism.

Reporting on sex and gender	- Sex was confirmed through molecular cytogenetic analyses (CGH data).<br>- For the MSK MetTropism and TCGA cohorts, data were obtained from cBioPortal (https://www.cbioportal.org) and the GDC Portal (https://portal.gdc.cancer.gov).<br>- Basic demographics of the cohorts are provided in Supplementary Tables 1, 3, and 4.
Reporting on race, ethnicity, or other socially relevant groupings	Information on race, ethnicity, or other socially relevant groupings was not collected for the CRCTropism cohort.
Population characteristics	The age distributions (primary CRC and metastases) are provided in Supplementary Table 1. Information on treatment procedures was limited to surgical intervention and the exclusion of targeted therapy. Other demographic characteristics were not available. Basic demographics of the cohorts are listed in Supplementary Tables 3 (MSK MeTropism cohort) and 4 (TCGA cohort).
Recruitment	This study employs a retrospective design and reuses data from previously published studies.
Ethics oversight	The study was approved by the local ethics committees (University Medical Center Göttingen, Ludwig Maximilian University of Munich) and included data published previously. For the original data, the ethics committee of the University Medical Center Göttingen waived the requirement for informed consent. Data collection from the MSK MetTropism and TCGA (PanCancer Atlas) cohorts were collected by their respective research teams and made publicly available. The original studies reported that data collection was approved by their respective ethics committees, with informed consent obtained from patients.

Note that full information on the approval of the study protocol must also be provided in the manuscript.

Field-specific reporting

Please select the one below that is the best fit for your research. If you are not sure, read the appropriate sections before making your selection.

☒ Life sciences ☐ Behavioural & social sciences ☐ Ecological, evolutionary & environmental sciences

For a reference copy of the document with all sections, see nature.com/documents/nr-reporting-summary-flat.pdf

Life sciences study design

All studies must disclose on these points even when the disclosure is negative.

Sample size	No sample size was predefined; inclusion was based on the availability of cases/material.<br><br>Our study used three datasets available in CRC research:

CRCTropism Cohort:
- Largest brain metastases cohort untreated by targeted therapy
- dedicated molecular cytogenetics approach

MSKTropism Cohort:
- Largest Next-Generation Sequencing (NGS) based metastasis cohort
- Encompasses both primary CRC and metastatic lesions

TCGA-COAD/READ Cohort:
- Multi-omics characterized dataset of primary CRC
- Integrates genomic, transcriptomic, and proteomic data

This tri-cohort approach allows for cross-validation of findings, exploration of site-specific genomic alterations, and integration of multi-omic data, enhancing the depth and breadth of our analysis in CRC progression and metastasis.

Data exclusions	Quality criteria were established for tissue, DNA extracted from FFPE samples, and data collection. Processing of samples that did not meet quality standards was repeated at least once, provided that further material was available for re-analysis. If the samples still did not meet the quality criteria or if additional material was not available, those samples were excluded.
Replication	Three independent cohorts were analyzed using two different methods: molecular cytogenetics and next-generation sequencing (NGS). For research questions where multiple cohorts were applicable, we consistently reproduced the results across these independent datasets.
Randomization	Randomization was not relevant for this study because the type of lesion (primary tumor, liver metastases, lung metastases, brain metastases) defined the assignment to the respective groups.
Blinding	No blinding could be used because the data were also utilized for diagnostics. Patient outcomes were not assessed in this study.

Reporting for specific materials, systems and methods

We require information from authors about some types of materials, experimental systems and methods used in many studies. Here, indicate whether each material, system or method listed is relevant to your study. If you are not sure if a list item applies to your research, read the appropriate section before selecting a response.

Materials & experimental systems

n/a	Involved in the study	
☒	☐	Antibodies
☒	☐	Eukaryotic cell lines
☒	☐	Palaeontology and archaeology
☒	☐	Animals and other organisms
☒	☐	Clinical data
☒	☐	Dual use research of concern
☒	☐	Plants

Methods

n/a	Involved in the study	
☒	☐	ChIP-seq
☒	☐	Flow cytometry
☒	☐	MRI-based neuroimaging

Plants

Seed stocks	*Report on the source of all seed stocks or other plant material used. If applicable, state the seed stock centre and catalogue number. If plant specimens were collected from the field, describe the collection location, date and sampling procedures.*
Novel plant genotypes	*Describe the methods by which all novel plant genotypes were produced. This includes those generated by transgenic approaches, gene editing, chemical/radiation-based mutagenesis and hybridization. For transgenic lines, describe the transformation method, the number of independent lines analyzed and the generation upon which experiments were performed. For gene-edited lines, describe the editor used, the endogenous sequence targeted for editing, the targeting guide RNA sequence (if applicable) and how the editor was applied.*
Authentication	*Describe any authentication procedures for each seed stock used or novel genotype generated. Describe any experiments used to assess the effect of a mutation and, where applicable, how potential secondary effects (e.g. second site T-DNA insertions, mosiacism, off-target gene editing) were examined.*
